# The Axin2-snail axis promotes bone invasion by activating cancer-associated fibroblasts in oral squamous cell carcinoma

**DOI:** 10.1186/s12885-020-07495-9

**Published:** 2020-10-12

**Authors:** Yin-Zhe An, Eunae Cho, Junqi Ling, Xianglan Zhang

**Affiliations:** 1 Key laboratory of Oral Medicine, Guangzhou Institute of Oral Disease, Affiliated Stomatology Hospital of Guangzhou Medical University, Guangzhou, Guangdong China; 2 Department of Oral Pathology, Yonsei University College of Dentistry, Seoul, Republic of Korea; 3 BK21 PLUS Project, Yonsei University College of Dentistry, Seoul, South Korea; 4 Oral Cancer Research Institute, Yonsei University College of Dentistry, Seoul, Republic of Korea; 5 Department of Endodontics, Guanghua School of Stomatology, Guangdong Provincial Key Laboratory of Stomatology, Sun Yat-Sen University, 56 Lingyuanxi Road, Guangzhou, 510055 Guangdong China; 6 Department of Pathology, Yanbian University Hospital, Yanji City, 133000 Jilin Province China

**Keywords:** Axin2, Snail, Cytokine, Cancer-stroma crosstalk, CAFs, Bone invasion, Prognosis, OSCC

## Abstract

**Background:**

In bone-invasive oral squamous cell carcinoma (OSCC), cancer-associated fibroblasts (CAFs) infiltrate into bony tissue ahead of OSCC cells. In the present study, we aimed to investigate the role of the Axin2-Snail axis in the biological behaviour of CAFs and bone invasion in OSCC.

**Methods:**

The clinicopathological significance of Axin2 and Snail expression was investigated by immunohistochemistry in an OSCC cohort containing 217 tissue samples from patients with long-term follow-up. The influence of the Axin2-Snail axis on the biological behaviour of OSCC cells and CAFs was further investigated both in vitro and in vivo.

**Results:**

Axin2 expression was significantly associated with Snail expression, the desmoplasia status, and bone invasion in patients with OSCC. In multivariate analysis, lymph node metastasis, desmoplasia, Axin2 expression, and Snail expression were independent poor prognostic factors in our cohort. Consistent with these findings, OSCC cells demonstrated attenuated oncogenic activity as well as decreased expression of Snail and various cytokines after Axin2 knockdown in vitro. Among the related cytokines, C-C motif chemokine ligand 5 (CCL5) and interleukin 8 (IL8) demonstrated a strong influence on the biological behaviour of CAFs in vitro. Moreover, both the desmoplastic reaction and osteolytic lesions in the calvaria were predominantly decreased after Axin2 knockdown in OSCC cells in vivo using a BALB/c athymic nude mouse xenograft model.

**Conclusions:**

Oncogenic activities of the Axin2-Snail axis are not limited to the cancer cells themselves but rather extend to CAFs via regulation of the cytokine-mediated cancer-stromal interaction, with further implications for bone invasion as well as a poor prognosis in OSCC.

## Background

Oral squamous cell carcinoma (OSCC) is the most common histological type of oral cancer. OSCC cells often penetrate underlying bone, and 12–56% of patients with OSCC present with bone invasion [1]. According to the American Joint Committee on Cancer (AJCC) classification, the presence of bone invasion can upstage this type of cancer regardless of tumour size because bone invasion is a major poor prognostic indicator of OSCC [2–4]. However, the molecular mechanism underlying the invasion of adjacent bone by OSCC is not fully understood.

Desmoplasia refers to the growth of excessive stromal tissue around tumours, and fibroblasts located in this stromal tissue, termed cancer-associated fibroblasts (CAFs), are key players in the cancer stroma. The critical roles of CAFs have been investigated recently in various types of cancer [5–8]. CAFs promote angiogenesis via the production of pro-angiogenic factors such as fibroblast growth factor 2 (FGF2) and vascular endothelial growth factor A (VEGFA) and contribute to immune surveillance in tumour cells by recruiting immunosuppressive cells such as myeloid-derived suppressor cells (MDSCs) and M2 macrophages [9–11]. Moreover, CAF-derived hepatocyte growth factor (HGF), stromal cell-derived factor 1 (SDF1), and transforming growth factor beta (TGF-β) promote the proliferation, invasion, and metastasis of cancer cells by activating various signalling pathways, such as the MAPK and PI3K/AKT pathways, as well as the TGF-β/SMAD pathway [12–14]. In bone-invasive OSCC, an abundant population of stromal cells intervenes between OSCC cells and damaged bony tissue. In particular, alpha-smooth muscle actin (α-SMA)-positive CAFs, a major component of the cancer-related stroma, have been shown to infiltrate into bony tissue ahead of OSCC cells [15]. The biological characteristics of CAFs may be important in the pathogenesis of bone invasion in OSCC.

CAFs demonstrate heterogeneity in tumour tissues. In pancreatic adenocarcinomas, for example, CAFs display two distinct populations, myofibroblasts and inflammatory fibroblasts, while in breast cancer, four different populations of CAFs are observed, including those related to immune escape [16, 17]. Moreover, studies on various cancers, including OSCC, have revealed the presence of a senescence-associated secretory phenotype of CAFs that can secrete various cytokines and thereby influence the tumour microenvironment [18–20]. The stromal role may differ according to the particular population of CAFs.

The production as well as transdifferentiation of CAFs may be regulated by cancer-stromal crosstalk. Some tumour-derived factors, particularly proinflammatory cytokines, trigger cancer-stromal crosstalk and further influence multifaceted signalling pathways responsible for cancer progression, thereby promoting the formation of a favourable microenvironment for tumour cell survival, growth, and invasion [21, 22]. Recently, therefore, CAFs have become regarded as a potential target to increase the therapeutic efficacy of the treatment of various cancers with desmoplastic features [23]. Accordingly, targeting the key genetic factors involved in the desmoplastic reaction in cancers may enable therapeutic advances for patients resistant to current anti-cancer therapy.

Epithelial to mesenchymal transition (EMT) is mediated by local activation of the canonical Wnt signalling pathway in various types of cancer and promotes cell invasion and metastasis during cancer progression by silencing epithelial traits and inducing mesenchymal phenotypes [24]. On the invasive front, cancer cells are exposed to the extracellular matrix and can thereby participate in cancer-stromal crosstalk. Snail, a zinc-finger transcription factor, is primarily known as a transcriptional repressor that mediates EMT via the repression of E-cadherin transcription [25–27]. Recently, increasing evidence has demonstrated that Snail-mediated transactivation can remodel the tumour microenvironment during EMT progression. The transcription of multiple proinflammatory cytokines, such as C-C motif chemokine ligand (CCL) 2 and CCL5, is activated by Snail transactivation [28, 29]. Moreover, Snail can also directly activate interleukin (IL) 8 transcription by binding to the E3/E4 E-boxes [30]. In addition, Snail activates TGF-β signalling pathway-related proteins, such as connective tissue growth factor, secreted protein acidic and rich in cysteine, fibronectin 1, and transgelin, which are involved in fibrosis in various organs. Axis inhibition protein 2 (Axin2), a scaffolding protein of glycogen synthase kinase 3 (GSK-3), promotes cancer cell invasion and metastasis in various types of malignancies by inhibiting GSK-3-mediated Snail degradation [25, 31]. Axin2-mediated Snail stabilization may contribute to cancer-stromal crosstalk and thereby may influence cancer prognosis.

In the present study, the association between Axin2 expression and various clinicopathological factors, including the desmoplasia status as well as bone invasion, was investigated in patients with OSCC. The influence of Axin2 expression on the biological behaviour of CAFs was also investigated in vitro and in vivo with the aim of identifying the possible role of Axin2 expression in the pathogenesis of OSCC.

## Methods

### Patients in the OSCC cohort

**Fig. 1:**
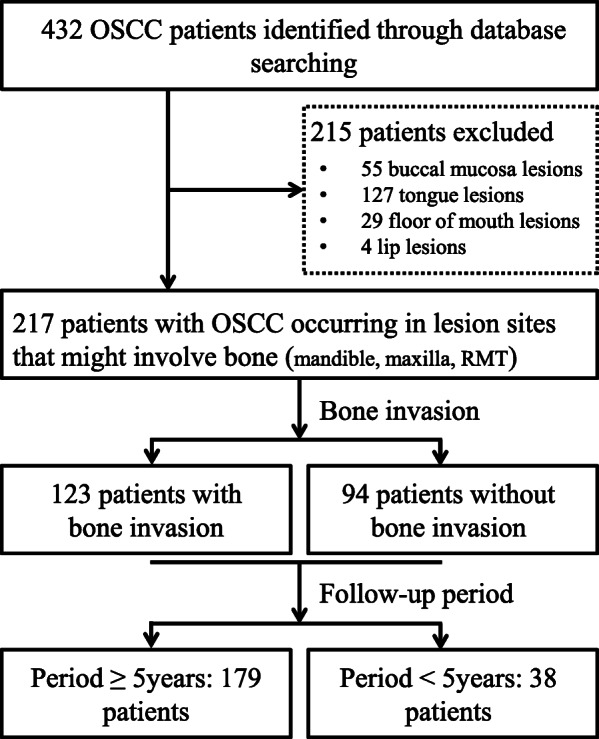
Flow diagram of sample selection and enrolment for patients with OSCC

In this study, we retrospectively reviewed the archived files of patients with OSCC at Dental Hospital, Yonsei University Medical Center, Seoul, Korea, from 1999 to 2017. Of the 432 patients reviewed, 217 with OSCC occurring in lesion sites that might involve the maxilla and/or the mandible were included in the present study. Of these 217 patients in the OSCC cohort, 123 (56.7%) had been determined during follow-up to have bone invasion, while no bone invasion had been found in the other 94 (43.3%) patients (Fig. 1). The clinicopathological characteristics of the patients in the OSCC cohort are shown in Table 1. This study was approved by the Institutional Review Board for Bioethics of the Yonsei University College of Dentistry (IRB 2–2017-0006 and 2–2019-0050).

**Table 1:** Clinicopathological characteristics of 217 OSCC patients

Clinicopathological variables	No. of patients (%)
Total cases	217
Age, years	
Median age (range)	61 (27–85)
≤ 61	113 (52.1)
> 61	104 (47.9)
Sex	
Male	141 (65.0)
Female	76 (35.0)
Site	
Mandible	112 (51.6)
Maxilla	67 (30.9)
RMT	38 (17.5)
T stage	
T1	27 (12.4)
T2	45 (20.7)
T3	9 (4.1)
T4	136 (62.7)
N stage	
Nx	48 (22.1)
N0	95 (43.8)
N1	23 (10.6)
N2a	9 (4.1)
N2b	25 (11.5)
N2c	1 (0.5)
N3b	16 (7.4)
Histologic grade	
WD	35 (16.1)
MD	149 (68.7)
PD	33 (15.2)
Perineural invasion	
Negative	202 (93.1)
Postive	15 (6.9)
Vascular invasion	
Negative	192 (88.5)
Postive	25 (11.5)
Bone invasion	
Negative	94 (43.3)
Postive	123 (56.7)

*WD* well differenciated, *MD* moderately differentiated, *PD* poorly differentiated

### Immunochemical staining

The protein expression of α-SMA, vimentin, CD31, Axin2, and Snail in cells and tissue samples was determined by immunochemical staining. Details on the procedures and scoring methods of immunochemical staining are described in the Supplementary Materials and Methods.

### Histomorphometry

As described in a previous study, the desmoplastic reaction status was evaluated according to the ratio of the area of the tumour-associated stroma to that of the whole tumour tissue [32]. Histologic images were obtained using the light microscope (Olympus BX53®, Tokyo, Japan) with an attached digital camera (DP27®, Japan) and Olympus cellSens™ Entry software. The subjected areas were measured on haematoxylin and eosin-stained OSCC tumour tissue sections with ImageJ software. The desmoplasia status was further divided into two groups according to the calculated ratio: high (ratio ≥ 1) and low desmoplastic (ratio < 1) reaction.

### Cell lines

Two kinds of CAFs (CAF1 and CAF2) and two OSCC cell lines (CA9–22 and HSC-2) were used in this study. Details on the procedures of cell culture and the establishment of Axin2-knockdown OSCC cells are described in the Supplementary Materials and Methods.

### Recombinant proteins

All of the human recombinant proteins (CCL2, CCL5, and IL8) were purchased from R&D Systems (Minneapolis, MN, USA).

### Proliferation assay

To investigate the proliferative ability of each group of cells, OSCC cells (1 × 10^5^ /well) and CAFs (5 × 10^4^ /well) were seeded into 6-well plates at approximately 30% confluency. The number of cells was counted after trypan blue staining at each indicated time point.

### Wound healing and invasion assays

Cell motility and invasion ability were determined for each group of cells using wound healing and Matrigel invasion assays, respectively. Details on the procedures of wound healing and invasion assays are described in the Supplementary Materials and Methods.

### Quantitative reverse transcription polymerase chain reaction (RT-PCR)

Quantitative RT-PCR was performed to determine the mRNA expression of IL8, CCL2, CCL5, matrix metalloproteinase (MMP)-2, MMP-9, and Ki67 in the cell lines. Details on the RT-PCR procedure are described in the Supplementary Materials and Methods.

### Nude mouse xenograft model

The animal studies were performed according to experimental protocols approved by the Animal Ethics Committee of the Yonsei University College of Dentistry. Twenty female 5-week-old BALB/c athymic nude mice (Central Laboratory Animal Inc., Seoul, Korea) were housed in laminar flow cabinets under specific pathogen-free conditions.

CAFs are derived from resident fibroblasts, bone marrow-derived mesenchymal precursor cells, adipocytes, endothelial cells, and EMT, endothelial to mesenchymal transition (EndMT), and various cytokines are implicated in the production of CAFs [33]. As well-known EMT genes, those involved in the Axin2-Snail axis may also contribute to the production of CAFs. In this study, we aimed to investigate the influence of Axin2 knockdown on both the production and biological behaviour of CAFs using a xenograft mouse model.

All of the mice were randomized into four groups (*n* = 5 per group), and a total of 1 × 10^6^ HSC-2^Mock^, HSC-2^△Axin2^, CA9–22^Mock^, and CA9–22^△Axin2^ cells were subcutaneously injected into the calvaria of the mice. The length and width of the tumour nodules were measured every 3 days, and the size of the tumours was calculated with the following formula: width^2^ × length× 1/2. We have acquired micro-computed tomography (micro-CT) images with Skyscan 1173 (Bruker-microCT, Kontich, Beigium) and reconstructed those images with Nrecon software. All of the mice were sacrificed after 9 weeks by CO_2_ asphyxiation.

### Statistical analysis

The association between protein expression and clinicopathological variables was analysed using the chi-square test. The Mann-Whitney U-test was used to compare proliferation, migration, invasion, and tumorigenesis between groups. SPSS software version 23.0 (SPSS Inc., Chicago, IL, USA) was used for statistical analysis, and a *p*-value less than 0.05 was considered statistically significant.

## Results

### Clinicopathological significance of Axin2 and snail expression in patients with OSCC

**Fig. 2:**
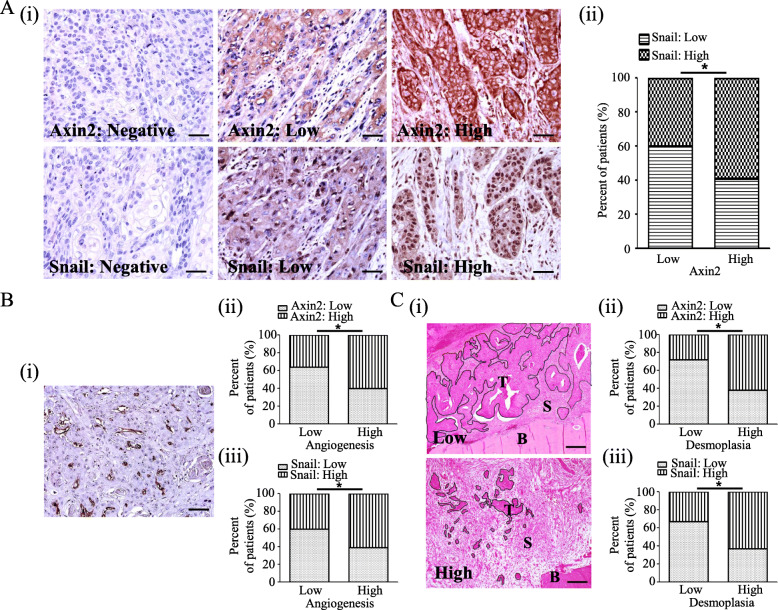
Clinicopathological significance of Axin2 and Snail expression in patients with OSCC. **a** Representative expression patterns for Axin2 and Snail in OSCC tissue samples (original magnification, × 400; scale bar, 25 μm) (i). Axin2 expression and Snail expression are significantly correlated in OSCC tissues (ii). **b** Association between microvessel density and the expression of Axin2 and Snail in OSCC tissues: Example of a hot spot in OSCC tissues (original magnification, × 200; scale bar, 50 μm) (i). Microvessel density is significantly associated with Axin2 and Snail expression (ii-iii). **c** Association between the desmoplastic reaction and the expression of Axin2 and Snail in OSCC tissues: representative histological patterns of low and high desmoplastic reactions in OSCC tissues (i). The desmoplastic reaction is significantly associated with Axin2 and Snail expression (original magnification, × 100; scale bar, 100 μm) (ii-iii) (* *p* < 0.05) (T: tumour, S: stroma, B: bone)

In the present study, Axin2 expression was found in the cytoplasm of cancer cells in 168 (77.4%) patients with OSCC, and immunoreactivity against Axin2 was high in 101 (high-Axin2, 46.5%) OSCC tissue samples and low in 116 (low-Axin2, 53.5%). Cancer cells demonstrated cytoplasmic and nuclear Snail expression in 186 (85.7%) patients with OSCC, and immunoreactivity against Snail was high in 107 (high Snail, 49.3%) OSCC tissue samples and low in 110 (low Snail, 50.7%). A significant association was found between Axin2 expression and Snail expression in patients with OSCC (*p* = 0.006) (Fig. 2a). Both high Axin2 expression and high Snail expression showed a significant association with T stage (*p* < 0.001 and *p* = 0.031), lymph node metastasis (both *p* < 0.001), vascular invasion (*p* = 0.01 and *p* = 0.019), and bone invasion (*p* < 0.001 and *p* = 0.028) in the present study. The sex differences in the prognosis of OSCC patients remain controversial [34]. In our study, we did not find a significant association between sex and the prognosis of OSCC. Moreover, there was no significant association between sex and Axin2 or Snail expression in our cohort. The current edition of the “Classification of Head and Neck Tumors” suggests three histological grades for conventional OSCC: well-, moderately, and poorly differentiated variant, in spite the fact that they remark grading as ‘Grading alone does not correlate well with prognosis’ [35]. Consistent with this, we did not find a significant association between histological grade and prognosis in our cohort. High Axin2 expression showed a tendency to gradually increase from well-differentiated OSCC (37.1%, 13/35) to moderately differentiated OSCC (47.7%, 71/149) to poorly differentiated OSCC (51.5%, 17/33). However, there were no significant differences between the groups (Table 2).

**Table 2:** Clinicopathological significance of Axin2 and Snail expression in 217 OSCC patients

Variables	Total	Axin2		*P*	Snail		*P*
		Low	High		Low	High	
Age							
≤ 61	113	65 (57.5)	48 (42.5)	0.211	60 (53.1)	53 (46.9)	0.46
> 61	104	51 (49.0)	53 (51.0)		50 (48.1)	54 (51.9)	
Sex							
Male	141	69 (48.9)	72 (51.1)	0.069	74 (52.5)	67 (47.5)	0.472
Female	76	47 (61.8)	29 (38.2)		36 (47.4)	40 (52.6)	
Site							
Mandible	112	66 (58.9)	46 (41.1)	0.173	60 (53.6)	52 (46.4)	0.681
Maxilla	67	34 (50.7)	33 (49.3)		32 (47.8)	35 (52.2)	
RMT	38	16 (42.1)	22 (57.9)		18 (47.4)	20 (52.6)	
T stage							
T1-T2	72	51 (70.8)	21 (29.2)	< 0.001	44 (61.1)	28 (38.9)	0.031
T3-T4	145	65 (44.8)	80 (55.2)		66 (45.5)	79 (54.5)	
N stage							
Nx	48	31 (64.6)	17 (35.4)	< 0.001	29 (60.4)	19 (39.6)	< 0.001
N0	95	62 (65.3)	33 (34.7)		57 (60.0)	38 (40.0)	
N1–3	74	23 (31.1)	51 (68.9)		24 (32.4)	50 (67.6)	
Histologic grade							
WD	35	22 (62.9)	13 (37.1)	0.439	19 (54.3)	16 (45.7)	0.565
MD	149	78 (52.3)	71 (47.7)		77 (51.7)	72 (48.3)	
PD	33	16 (48.5)	17 (51.5)		14 (42.4)	19 (57.6)	
Perineural invasion							
Negative	202	110 (54.5)	92 (45.5)	0.279	105 (52.0)	97 (48.0)	0.163
Postive	15	6 (40.0)	9 (60.0)		5 (33.3)	10 (66.7)	
Vascular invasion							
Negative	192	109 (56.8)	83 (43.2)	0.01	103 (53.6)	89 (46.4)	0.019
Postive	25	7 (28.0)	18 (72.0)		7 (28.0)	18 (72.0)	
Bone invasion							
Negative	94	63 (67.0)	31 (33.0)	< 0.001	56 (59.6)	38 (40.4)	0.028
Postive	123	53 (43.1)	70 (56.9)		54 (43.9)	69 (56.1)	

*WD* well differenciated, *MD* moderately differentiated, *PD* poorly differentiated

Moreover, patients with high Axin2 or high Snail expression demonstrated increased vessel density (*p* < 0.001 and *p* = 0.002) and a higher desmoplastic reaction (both *p* < 0.001) than patients with low Axin2 or low Snail expression in our cohort (Fig. 2b and c). To identify the risk factors for the prognosis of OSCC, multivariate analysis was performed in 179 patients who were followed up for more than 5 years. The results showed that when using age, sex, lesion site, T stage, lymph node metastasis, histologic grade, vascular invasion, perineural invasion, bone invasion, desmoplasia status, angiogenesis status, Axin2 expression, and Snail expression as cofactors, lymph node metastasis, desmoplasia status, Axin2 expression, and Snail expression were independent risk factors for OSCC prognosis, with hazard ratios of 3.424 (95% confidence interval, 1.466–7.998; *p* = 0.004), 2.491 (95% confidence interval, 1.240–5.004; *p* = 0.01), 2.488 (95% confidence interval, 1.358–4.559; *p* = 0.003), and 1.984 (95% confidence interval, 1.097–3.588; *p* = 0.024), respectively (Table 3).

**Table 3:** Prognostic impact of clinical variables and biomarkers in multivariate Cox regression analysis in 179 OSCC patients

	Hazard ratio (95% CI)	*p*
Age	0.825 (0.482–1.410)	0.481
Sex	0.609 (0.339–1.093)	0.096
Lesion site		
Mandible	1	0.235
Maxilla	0.618 (0.289–1.323)	0.215
RMT	1.206 (0.608–2.392)	0.591
T stage		
T1		0.99
T2	0.989 (0.319–3.066)	0.985
T3	1.059 (0.119–9.453)	0.959
T4	1.157 (0.386–3.467)	0.795
N stage		
Nx		0.014
N0	1.927 (1.016–3.655)	0.044
N1–3	3.424 (1.466–7.998)	0.004
Histologic grade		
WD	1	0.435
MD	1.209 (0.592–2.467)	0.602
PD	1.665 (0.730–3.798)	0.226
Perineural invasion	0.850 (0.321–2.247)	0.743
Vascular invasion	1.036 (0.457–2.350)	0.933
Bone invasion	1.357 (0.572–3.219)	0.489
Desmoplasia	2.491 (1.240–5.004)	0.01
Angiogenesis	1.449 (0.860–2.441)	0.164
Axin2	2.488 (1.358–4.559)	0.003
Snail	1.984 (1.097–3.588)	0.024

*WD* well differenciated, *MD* moderately differentiated, *PD* poorly differentiated, *95% CI* 95% confidence interval

### Axin2 knockdown had a strong influence on the biological behaviour of OSCC cells

Consistent with the results of a previous study [25], we found that Snail expression was predominantly decreased in both CA9–22^△Axin2^ and HSC-2^△Axin2^ cells compared to the related control cells (Supplementary Fig. A, i and iv).

Proliferative ability was significantly reduced after Axin2 knockdown in both CA9–22 and HSC-2 cells. Compared with CA9–22^Mock^ cells, decreases of 1.4-, 2.1-, and 2.2-fold in cell number were found in CA9–22^△Axin2^ cells after 24 h, 48 h, and 72 h of culture (all *p* = 0.008). Similarly, HSC-2^△Axin2^ cells also showed decreases of 1.4-, 1.6-, and 2.3-fold in number compared to HSC-2^Mock^ cells (all *p* = 0.008) (Supplementary Fig. A, ii and v). Likewise, Ki67 expression was significantly decreased in Axin2-knockdown cells compared to both HSC-2 and CA9–22 control cells (both *p* = 0.002) (Supplementary Fig. A, iii and vi). In addition, cell motility was decreased 1.5- and 1.8-fold, respectively, in Axin2-knockdown cells compared to CA9–22 and HSC-2 control cells (both *p* = 0.002) (Supplementary Fig. B, i-iv). Moreover, 2.3- and 1.6-fold decreases in the numbers of invading cells were found in Axin2-knockdown cells compared to CA9–22 and HSC-2 control cells, respectively (both *p* = 0.002). Axin2 may have oncogenic activity in OSCC cells (Supplementary Fig. C, i-iv). Interestingly, compared to the related control cells, the expression of Snail-related cytokines IL8, CCL2, and CCL5 was 3.5-fold, 2.8-fold, and 3.3-fold decreased, respectively, in CA9–22^△Axin2^ cells (all *p* = 0.002) and 2.6-fold, 1.8-fold, and 1.5-fold decreased, respectively, in HSC-2^△Axin2^ cells (all *p* = 0.002) (Supplementary Fig. D, i-ii).

### Cytokines related to the Axin2-snail axis exert strong influences on the biological behaviour of CAFs

**Fig. 3:**
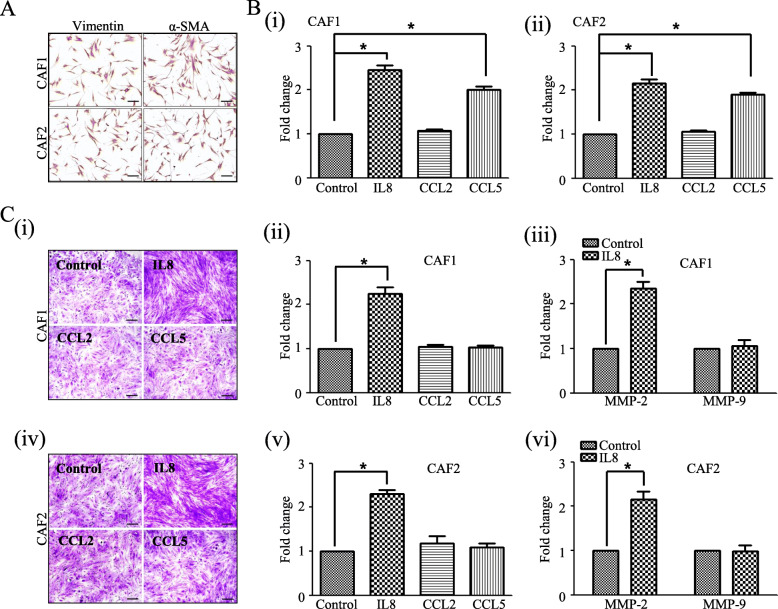
Cytokines related to the Axin2-Snail axis exert strong influences on the biological behaviour of cancer-associated fibroblasts (CAFs) in vitro. **a** CAF identities were verified based on the expression of vimentin and α-SMA. **b** Proliferation was significantly increased after CCL5 or IL8 treatment in both CAF1 and CAF2 cells (i & ii). **c** Invasion ability and MMP-2 expression were significantly increased after IL8 treatment in both CAF1 (i-iii) and CAF2 (iv-vi) cells (original magnification, × 100; scale bar, 100 μm) (* *p* < 0.05)

To evaluate the effect of these cytokines on the biological behaviour of CAFs, both CAF1 and CAF2 cells were treated with different doses (0, 2, 5, and 10 ng/ml) of human recombinant proteins (IL8, CCL2, and CCL5), after which the proliferation and invasion abilities of CAFs in each group were comparatively investigated. A strong influence of IL8 or CCL5 on the biological behaviour of CAFs was found at a dose of 2 ng/ml in the present study. CAF1 cells showed increases of 2.5- and 2.0-fold in the numbers of cells after treatment with 2 ng/ml IL8 or CCL5, respectively, compared to untreated control cells (both *p* = 0.002). Similar results were obtained using CAF2, with IL8 or CCL5 treatment leading to increases in cell numbers (both *p* = 0.002). No significant differences were observed in the proliferative ability of CAFs after CCL2 treatment in this study (Fig. 3b, i-ii).

We also found that the invasion ability of CAF1 and CAF2 cells was 2.2- and 2.3-fold increased, respectively, after IL8 (2 ng/ml) treatment compared to untreated control cells (both *p* = 0.002). Consistent with these findings, MMP-2 expression was 2.3-fold and 2.2-fold increased after IL8 (2 ng/ml) treatment in both CAF1 and CAF2 cells, respectively, compared to untreated control cells (both *p* = 0.002). No significant difference was found in MMP-9 expression in CAFs after IL8 treatment in our study (Fig. 3c, i-vi).

### Tumour progression and bone invasion depend on Axin2 expression in tumour cells in vivo

**Fig. 4:**
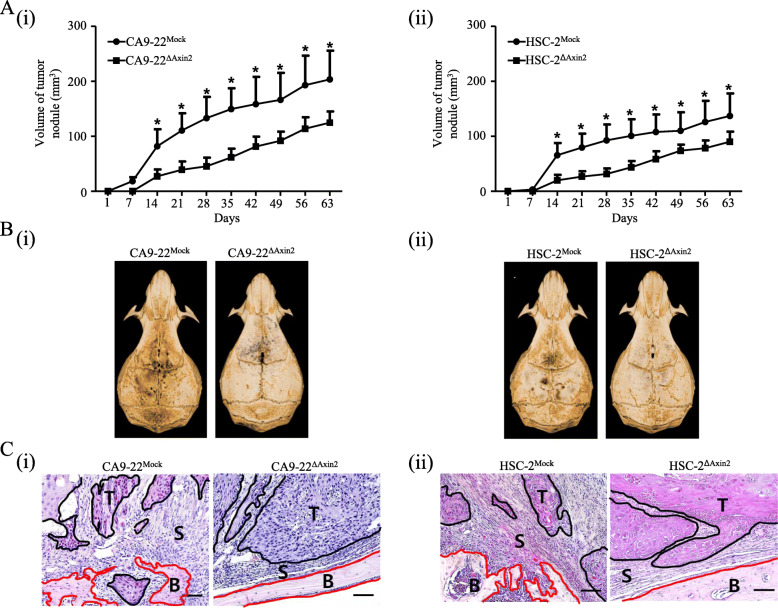
Tumour progression and bone invasion depend on Axin2 expression in tumour cells in vivo. **a** Tumour volume was predominantly decreased in mice injected with Axin2-knockdown cells compared to both HSC-2 (i) and CA9–22 control cells (ii). **b** In micro-CT imaging analysis revealed extensive osteolytic lesions in the calvaria from HSC-2^Mock^ or CA9–22^Mock^ cell-bearing mice compared to the related Axin2-knockdown cell-bearing mice (i-ii). **c** The area of the tumour-associated stroma was predominantly increased at the tumour-bone interface in CA9–22^Mock^ or HSC-2^Mock^ cell-bearing mice compared to the related Axin2-knockdown cell-bearing mice (i-ii) (original magnification, × 200; scale bar, 50 μm, T: tumour, S: stroma, B: Bone) (* *p* < 0.05)

As shown in Fig. 4, tumour volume was significantly decreased in mice injected with Axin2-knockdown cells compared to both CA9–22 and HSC-2 control cells (A, i-ii). In micro-CT imaging analysis, extensive osteolytic lesions were observed in the calvaria from CA9–22^Mock^ or HSC-2^Mock^ cell-bearing mice compared to the related Axin2-knockdown cell-bearing mice (B, i-ii). Moreover, in the tissue sections, the area of the tumour-associated stroma was predominantly increased at the tumour-bone interface in CA9–22^Mock^ or HSC-2^Mock^ cell-bearing mice compared to the related Axin2-knockdown cell-bearing mice (C, i-ii).

## Discussion

Although earlier studies focused on the inhibitory role of Axin2 in the β-catenin degradation complex, recent studies have implied that Axin2 contributes to oncogenic progression. Increased expression of Axin2 was found after the loss of adenomatous polyposis coli (APC), a key tumour suppressor gene, in colorectal cancer, and the knockdown of Axin2 attenuated oncogenic and Wnt signalling activities in cancer cells [36, 37]. The GSK3 nuclear export function of the Axin2-mediated abundance of nuclear Snail and β-catenin is also a sign of the oncogenic activity of Axin2 [25]. In our previous study, we found that Axin2 expression showed a positive correlation with Snail expression, and increased expression of both Axin2 and Snail was closely associated with the malignant transformation of oral leukoplakia [38]. Consistent with these findings, high Axin2 expression and high Snail expression are significantly correlated in OSCC tissues. Moreover, we found that both Axin2 expression and Snail expression were significantly related to a poor prognosis and serve as poor prognostic indicators of OSCC, including bone invasion in OSCC. Consistent with these results, Axin2-knockdown cells showed decreased Snail expression and attenuated oncogenic activities compared to control cells. Axin2 may be implicated in OSCC pathogenesis as an oncogene.

Desmoplasia is a typical sign of aggressiveness in various types of cancer, including OSCC [15, 39]. Some investigators have shown that an abundant desmoplastic reaction is prominent in the cancer-bone interface of OSCC with bone invasion, but an identifiable fibrous stroma has been less frequently found in ameloblastoma, a benign odontogenic epithelial tumour with features of bone invasion [15]. In the present study, we also found frequent desmoplastic reactions in OSCC tissues, especially in the area of bone invasion, and the desmoplasia status was significantly associated with bone invasion in patients with OSCC. Interestingly, both Axin2 expression and Snail expression were positively correlated with the desmoplastic reaction in our cohort. Moreover, in the mouse xenograft analysis, the stromal component of tumour nodules as well as the osteolytic bone resorption of mouse calvaria were predominantly decreased in the group of cells with Axin2 knockdown compared to control cells. In addition, the expression of Snail-mediated proinflammatory cytokines, such as IL8, CCL2, and CCL5, was significantly decreased after Axin2 knockdown in OSCC cells compared to control cells. Admittedly, all of these cytokines are related to characteristics of aggressiveness by means of promoting the migration, invasion, and metastatic abilities of cancer cells, including those of OSCC, and are considered potential therapeutic targets of malignancies [40–44].

According to previous studies, all of these tumour-derived cytokines also influence different types of cells in the stromal component of tumours, such as CAFs, endothelial cells, and inflammatory cells, and thereby trigger multiple signalling pathways related to the malignant progression of tumours. Both CCL2 and CCL5 mediate the infiltration of tumour-associated macrophages and inhibit potential anti-tumour T-cell activities, thereby controlling the populations of leukocytes at tumour sites [43]. Moreover, distinct regulatory roles as well as underlying molecular mechanisms performed by CCL2 and IL8 in angiogenesis have been indicated by some investigators. CCL2 enhances angiogenic activity either by directly inducing endothelial cell retraction or by the CCL2-induced release of angiogenic factors such as VEGFA [45, 46]. IL8 promotes the invasion ability of endothelial cells via increased expression of MMP-2 and MMP-9 in endothelial cells [47]. Moreover, IL8 promotes the proliferation and tube formation of endothelial cells by activating extracellular signal-regulated protein kinase 1/2 (Erk1/2) during interaction with CXCR2 in endothelial cells [48]. In the present study, we found that CD31-positive vessel density was significantly associated with Axin2 and Snail expression in patients with OSCC. In OSCC, the Axin2-Snail axis may also mediate angiogenic responses via the control of related cytokines.

A previous study demonstrated that 12 different types of chemokine receptors were found in oral fibroblasts, including CXCR1, CXCR2, and CCR3 [49]. In the present study, we found that both CCL5 and IL8 have a strong influence on the biological behaviour of CAFs. These cytokines may influence CAFs by binding to related receptors. All of these findings imply that the Axin2-Snail axis mediates a diffuse desmoplastic reaction in OSCC by controlling inflammation-stromal crosstalk.

In the present study, recombinant IL8 increased the invasion ability of CAFs in vitro*.* In various types of cancer tissues, IL8 participates in degradation of the extracellular matrix by promoting the expression of MMP-2 and MMP-9 in cancer cells [50–53]. According to an analysis of a public data base, MMP-9 acts in a context-dependent manner in different types of cancer [54]. In this study, no significant difference was found in MMP-9 expression in CAFs after IL8 treatment. In the present study, we also found that MMP-2 expression was significantly increased in CAFs after IL8 treatment. A previous study showed that MMP-2 was expressed primarily in the fibroblasts of mouse lung tumours and concluded that CAFs were the main producer of MMP-2 [55]. MMPs can directly destroy the bone matrix; thus, IL8-mediated MMP-2 overexpression may be one of the underlying molecular mechanisms of CAFs infiltration into bony tissue ahead of OSCC cells in patients with high Axin2 expression.

## Conclusions

Consistent with previous observations in other cancers [25], our results imply that the Axin2-Snail axis is a poor prognostic indicator of OSCC. We also found that the oncogenic activities of the Axin2-Snail axis are not limited to the cancer cells themselves but rather extend to cancer-associated stromal cells such as endothelial cells and CAFs via regulation of the cytokine-mediated cancer-stromal interaction, thereby promoting active desmoplastic reactions as well as bone invasion in OSCC. The Axin2-Snail axis may serve as a novel diagnostic and therapeutic target in bone-invasive OSCC.

## Supplementary information

**Additional file 1.** Supplementary materials and methods

**Additional file 2: Figure S**. Influence of Axin2 knockdown on the biological behaviour of OSCC cell lines. (A) The expression of both Axin2 and Snail was strongly decreased after Axin2 knockdown in CA9–22 (i) and HSC-2 (iv) cell lines (original magnification, × 400; scale bar, 25 μm). Both the cell number and Ki67 mRNA expression were significantly decreased after Axin2 knockdown in CA9–22 (ii-iii) and HSC-2 cell lines (v-vi). Migration ability was significantly reduced after Axin2 knockdown in both CA9–22 (i-ii) and HSC-2 (iii-iv) cell lines (original magnification, × 200). (C) Invasion ability was significantly decreased after Axin2 knockdown in both CA9–22 (i-ii) and HSC-2 (iii-iv) cell lines (original magnification, × 100; scale bar, 100 μm). (D) IL8, CCL2, and CCL5 mRNA expression was significantly decreased after Axin2 knockdown in both CA9–22 (i) and HSC-2 (ii) cell lines (* *p* < 0.05)

**Additional file 3: Supplementary Table 1.** The primer sequences for quantitative reverse transcription polymerase chain reaction

## Data Availability

The datasets used and/or analysed during the current study are available from the corresponding author on reasonable request.

## Abbreviations

OSCC: Oral squamous cell carcinoma
CAFs: Cancer-associated fibroblasts
AJCC: American Joint Committee on Cancer
FGF2: Fibroblast growth factor 2
VEGFA: Vascular endothelial growth factor A
MDSC: Myeloid-derived suppressor cell
HGF: Hepatocyte growth factor
SDF1: Stromal cell-derived factor 1
TGF-β: Transforming growth factor beta
α-SMA: Alpha-smooth muscle actin
EMT: Epithelial to mesenchymal transition
CCL: C-C motif chemokine ligand
Axin2: Axis inhibition protein 2
GSK-3: Glycogen synthase kinase 3
RT-PCR: Reverse transcription polymerase chain reaction
MMP: Matrix metalloproteinase
EndMT: Endothelial to mesenchymal transition
micro-CT: Micro-computed tomography
APC: Adenomatous polyposis coli
Erk1/2: Extracellular signal-regulated protein kinase 1/2
