# Supplementary material for: The Axin2-snail axis promotes bone invasion by activating cancer-associated fibroblasts in oral squamous cell carcinoma
Source: BMC Cancer. 2020 Oct 12;20:987. doi: 10.1186/s12885-020-07495-9 (PMC7552517; doi:10.1186/s12885-020-07495-9)
Supplement: Supplementary file 1 — Additional file 1. Supplementary materials and methods [file 12885_2020_7495_MOESM1_ESM.docx]

***Supplementary data for***

The Axin2-Snail axis promotes bone invasion by activating cancer-associated fibroblasts in oral squamous cell carcinoma

Yin-Zhe An^1*^, Eunae Cho^2,3*^, Junqi Ling^1,4^, Xianglan Zhang^5,6^

^1^Key laboratory of Oral Medicine, Guangzhou Institute of Oral Disease, Affiliated Stomatology Hospital of Guangzhou Medical University, Guangzhou, Guangdong, China.

^2^Department of Oral Pathology, Yonsei University College of Dentistry, Seoul, Republic of Korea.

^3^BK21 PLUS Project, Yonsei University College of Dentistry, Seoul, Korea

^4^Department of Endodontics, Guanghua School of Stomatology, Guangdong Provincial Key Laboratory of Stomatology, Sun Yat-Sen University, Guangzhou, Guangdong, China.

^5^Department of Pathology, Yanbian University Hospital, Yanji, Jilin, China.

^6^Oral Cancer Research Institute, Yonsei University College of Dentistry, Seoul, Republic of Korea

* These two authors contributed equally.

**Correspondence to:**

Xianglan Zhang, Department of Pathology Yanbian University Hospital, Yanji City, Jilin Province,133000, China. E-mail address: zhangxianglan@yuhs.ac

Junqi Ling, Department of Endodontics, Guanghua School of Stomatology, Guangdong Provincial Key Laboratory of Stomatology, Sun Yat-Sen University, 56 Lingyuanxi Road, Guangzhou, Guangdong 510055, China. Email address: lingjq@mail.sysu.edu.cn

**Supplementary materials and methods**

Immunohistochemistry

In this study, mouse monoclonal anti-human α-SMA (Dako, Santa Clara, CA, USA), mouse monoclonal anti-human vimentin (Dako), rabbit monoclonal anti-human Axin2 (Abcam, Cambridge, UK), and rabbit polyclonal anti-human Snail [1] were used as primary antibodies for immunochemical staining.

Formalin-fixed paraffin-embedded OSCC tissue samples were cut into 4 μm tissue sections for immunohistochemistry. After deparaffinization and hydration, antigen retrieval was performed using antigen retrieval buffer (Dako). For immunocytochemistry, the cells were seeded into 6-well plates and fixed with 95% ethanol for 30 min at room temperature. Incubation with the primary antibodies was performed after blocking endogenous peroxidase activity with a mixture of methanol and hydrogen peroxide (ratio: 40:1), and the REAL EnVision HRP Rabbit/Mouse Detection System (Dako) was used as the secondary antibody. Visualization was performed with chromogen 3,3′-diaminobenzidine, and haematoxylin was used for counterstaining. For the negative control, the primary antibody was replaced with mouse or rabbit IgG (DakoCytomation Denmark A/S, Glostrup, Denmark). Histological images were acquired using the light microscope (Olympus BX53^®^, Japan) with an attached digital camera (DP27^®^, Japan) and Olympus cellSens™ Entry software.

As described in a previous study [2], the total histoscore of protein expression was analysed according to staining intensity and the percentage of positive cells. The patients were subdivided into two groups, a low expression group (total histoscore ≤100) and a high expression group (total histoscore>100), according to the total histoscore. For the scoring of vessel density, we selected the three areas with the highest CD31-positive vessel density on the slide, referred to as “hot spots”. In each hot spot, we further counted CD31-positive vessels under three fields at 200x magnification. The mean value of all CD31-positive vessels was considered the CD31-positive vessel density. The angiogenesis status was subdivided into two groups: low (vessel density ≤ mean vessel density of the cohort) and high (vessel density > mean vessel density of the cohort) angiogenic reactions.

OSCC cell culture and the establishment of Axin2-knockdown CA9-22 and HSC-2 cells

CA9-22 and HSC-2 cells were purchased from the Japanese Collection of Research Bioresources Cell Bank (Osaka, Japan) and cultured in medium containing a 3:1 ratio of Dulbecco’s modified Eagle’s medium (DMEM; Gibco BRL, Grand Island, NY, USA) and Ham’s F12 Nutrient Mixture (F12, Gibco BRL) supplemented with 10% foetal bovine serum (FBS; HyClone, Logan, UT, USA), 100 U/mL penicillin, 100 mg/mL streptomycin, 100 ng/mL cholera toxin, 0.4 μg/mL hydrocortisone, 5 μg/mL insulin, 5 μg/mL transferrin, and 2×10^−11^ M tri-iodothyronine.

Axin2-knockdown cells (CA9-22^△Axin2^ and HSC-2^△Axin2^) were constructed using the pLKO-Tet-On vector (Addgene, Cambridge, MA, USA) expressing a short hairpin RNA (shRNA) against Axin2 (target sequence: 5'-ACCACCACTACATCCACCA-3') (pLKO-Tet-shAxin2). Both CA9-22 and HSC-2 cells transfected with pLKO-Tet-shAxin2 were grown in culture media supplemented with 10% Tet-approved FBS (HyClone Laboratories Inc., Logan, UT, USA) and 1 μg/mL puromycin (CA9-22 ^Mock^ and HSC-2 ^Mock^). Axin2 shRNA expression was induced by 5 μg/mL doxycycline (Sigma-Aldrich, St. Louis, MO, USA), and the knockdown of Axin2 expression in each stable cell line was confirmed by immunocytochemical staining. Histological images were acquired using the light microscope (Olympus BX53^®^, Japan) with an attached digital camera (DP27^®^, Japan) and Olympus cellSens™ Entry software.

CAF culture from OSCC tissues

Primary CAFs were prepared using OSCC surgical specimens. The tissue samples were cut into approximately 2 mm^3^ cubes and cultured in DMEM (Gibco BRL, Grand Island, NY, USA) supplemented with 10% FBS (HyClone, Logan, UT, USA), 100 U/mL penicillin, and 100 mg/mL streptomycin. After 2 weeks of culture, the outgrown cells were harvested, and the identities of the cells were verified according to the expression of vimentin and α-SMA. The CAFs used in this study were at passage 4. This procedure was approved by the Institutional Review Board for Bioethics of the Yonsei University College of Dentistry (IRB 2-2012-0027).

Wound healing and invasion assays

For the wound healing assay, groups of OSCC cells and CAFs were seeded into 24-well plates at densities of 3x10^5^ and 1x10^5^ cells, respectively. Wound closure was determined 20 h after scratch wounding. The Transwell chamber culture system (BD Biosciences, Bedford, MA, USA) was used to quantify the invasion ability of the subjected cells. OSCC cells and CAFs were seeded onto the upper chamber of the transwell with culture medium containing 2% FBS at densities of 3x10^4^ and 1x10^4^, respectively. Culture medium containing 20% FBS was added to the lower chamber. After 36 h of incubation, penetrating cells were counted under a light microscope. Olympus BX53^®^ light microscope with an attached digital camera (DP27^®^, Japan) and Olympus cellSens™ Entry software were used for image analysis.

Quantitative RT-PCR

The mRNA expression levels of target genes were determined using 2× SYBR Premix Ex Taq II (Tli RnaseH Plus) (RR82LR; Takara, Ann Arbor, MI, USA) on an Applied Biosystems (Foster City, CA, USA) instrument by quantitative RT-PCR. Total RNA extraction was performed in each group of cells using TRIzol Reagent (Invitrogen), and oligo (dT) primers were used to synthesize complementary DNA. All of the mRNA expression levels were normalized to that of actin, and primer sequences are listed in Supplementary **Table 1**.

**Supplementary references**

1. Yook JI, Li XY, Ota I, Hu C, Kim HS, Kim NH, Cha SY, Ryu JK, Choi YJ, Kim J *et al*: **A Wnt-Axin2-GSK3beta cascade regulates Snail1 activity in breast cancer cells**. *Nat Cell Biol* 2006, **8**(12):1398-1406.

2. Zhang X, Zheng Z, Shin YK, Kim KY, Rha SY, Noh SH, Chung HC, Jeung HC: **Angiogenic factor thymidine phosphorylase associates with angiogenesis and lymphangiogenesis in the intestinal-type gastric cancer**. *Pathology* 2014, **46**(4):316-324.
