# Supplementary figures and images for: The Axin2-snail axis promotes bone invasion by activating cancer-associated fibroblasts in oral squamous cell carcinoma
Source: BMC Cancer. 2020 Oct 12;20:987. doi: 10.1186/s12885-020-07495-9 (PMC7552517; doi:10.1186/s12885-020-07495-9)

A

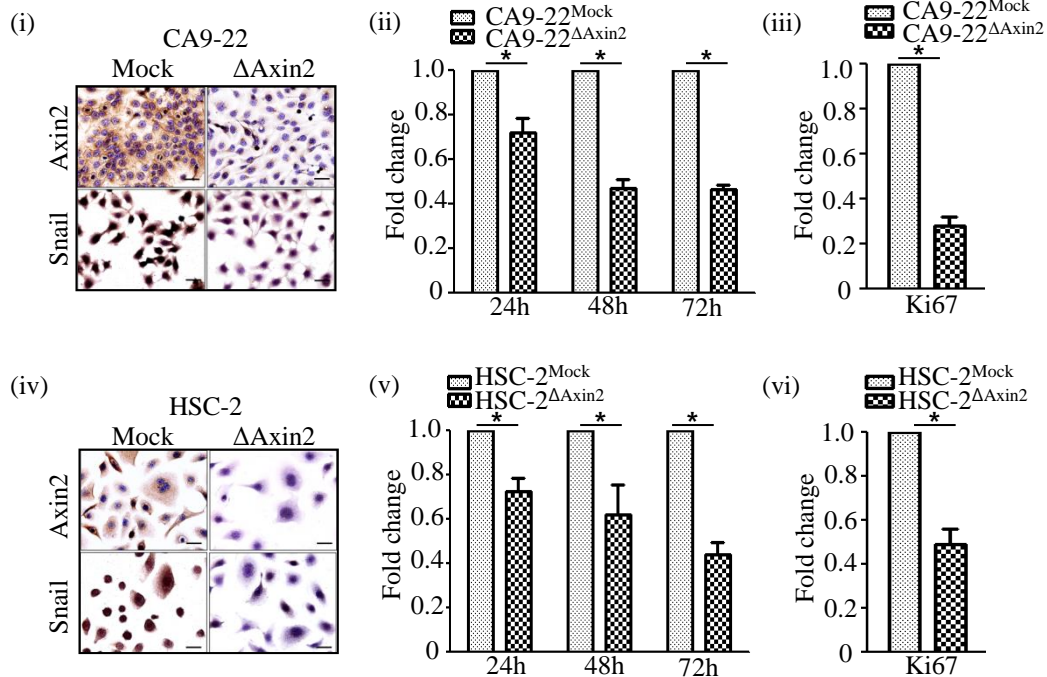

B

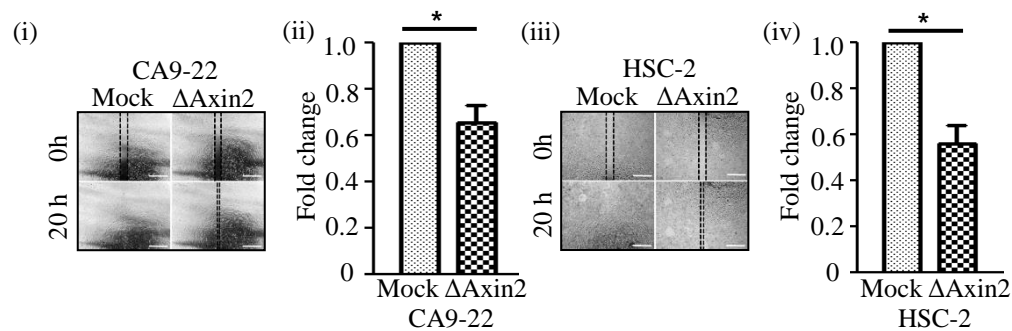

C

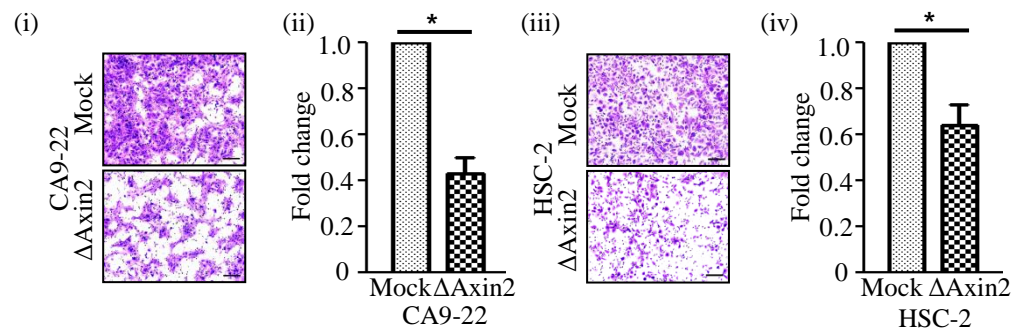

D

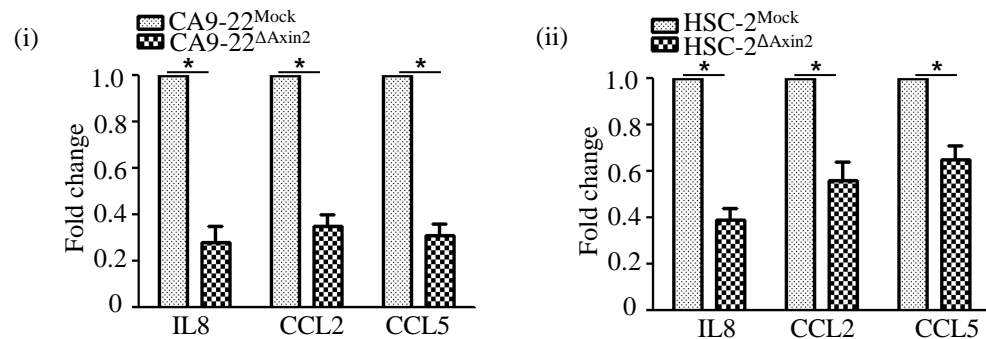

Supplement: Supplementary file 2 — Additional file 2: Figure S. Influence of Axin2 knockdown on the biological behaviour of OSCC cell lines. (A) The expression of both Axin2 and Snail was strongly decreased after Axin2 knockdown in CA9–22 (i) and HSC-2 (iv) cell lines (original magnification, × 400; scale bar, 25 μm). Both the cell number and Ki67 mRNA expression were significantly decreased after Axin2 knockdown in CA9–22 (ii-iii) and HSC-2 cell lines (v-vi). Migration ability was significantly reduced after Axin2 knockdown in both CA9–22 (i-ii) and HSC-2 (iii-iv) cell lines (original magnification, × 200). (C) Invasion ability was significantly decreased after Axin2 knockdown in both CA9–22 (i-ii) and HSC-2 (iii-iv) cell lines (original magnification, × 100; scale bar, 100 μm). (D) IL8, CCL2, and CCL5 mRNA expression was significantly decreased after Axin2 knockdown in both CA9–22 (i) and HSC-2 (ii) cell lines (* p < 0.05) [file 12885_2020_7495_MOESM2_ESM.pdf]
