# Supplementary material for: The Axin2-snail axis promotes bone invasion by activating cancer-associated fibroblasts in oral squamous cell carcinoma
Source: BMC Cancer. 2020 Oct 12;20:987. doi: 10.1186/s12885-020-07495-9 (PMC7552517; doi:10.1186/s12885-020-07495-9)
Supplement: Supplementary file 3 — Additional file 3: Supplementary Table 1. The primer sequences for quantitative reverse transcription polymerase chain reaction [file 12885_2020_7495_MOESM3_ESM.docx]

Supplementary Table 1. The primer sequences for quantitative reverse transcription polymerase chain reaction

| No. | Gene symbol | Sequence (5' to 3') |
| --- | --- | --- |
|  |  |  |
| 1 | IL8 | F: GTGCAGTTTTGCCAAGGAGT |
|  |  | R: CTCTGCACCCAGTTTTCCTT |
|  |  |  |
| 2 | CCL2 | F: AGT GTC CCA AAG AAG CTG TG |
|  |  | R:AGT TTG GGT TTG CTT GTC CA |
|  |  |  |
| 3 | CCL5 | F: CCA TAT TCC TCG GAC ACC AC |
|  |  | R:TTT CTT CTC TGG GTT GGC AC |
|  |  |  |
| 4 | MMP-2 | F: ATGACAGCTGCACCACTGAG |
|  |  | R:ATTTGTTGCCCAGGAAAGTG |
|  |  |  |
| 5 | MMP-9 | F: TTGACAGCGACAAGAAGTGG |
|  |  | R:GCCATTCACGTCGTCCTTAT |
|  |  |  |
| 6 | MKI67 | F: AAGCCCTCCAGCTCCTAGTC |
|  |  | R:TCCGAAGCACCACTTCTTCT |
|  |  |  |
| 7 | β-actin | F: GGACTTCGAGCAAGAGATGG |
|  |  | R: AGCACTGTGTTGGCGTACAG |

F: forward; R: reverse
